# How Personality, Emotions and Situational Characteristics Affect Learning from Social Interactions in the Workplace

**DOI:** 10.1007/s12186-022-09303-w

**Published:** 2022-11-10

**Authors:** Tamara Vanessa Leiß, Andreas Rausch

**Affiliations:** Mannheim Business School (MBS), University of Mannheim, Mannheim, Germany

**Keywords:** Workplace learning, Informal learning, Social interaction, Emotions, Personality, Diary study

## Abstract

The present study examines the effects of social interactions’ situational characteristics, emotions, and personality on self-perceived learning from social interactions at work based on diary and survey data. The sample comprises 43 German vocational education and training (VET) trainees in various apprenticeship programs. During the diary period of ten working days, the participants were instructed to record five typical social interactions at work every day. Quantitative data of 1,328 social interactions were analyzed by means of multilevel analysis. Regarding social interactions’ characteristics, the analysis revealed the baseline level of instrumentality, an interruption of the social interaction, its instrumentality and questions asked by the trainee during the interaction as positive predictors of self-perceived learning. A trainee’s higher speech proportion, however, was a negative predictor. Regarding state emotions, the emotional experiences of bored and motivated were identified as significant positive predictors of learning from social interactions at work. Emotions’ baseline level as well as personality traits had no significant influence. The results indicate that social interactions’ situational characteristics have the biggest influence on self-perceived learning from social interactions.

## Introduction

In this paper, we investigate how social interactions at work contribute to workplace learning and how situational characteristics of these interactions, emotions during these interactions, and personality traits are related to self-perceived learning from the interactions. The interest in workplace learning has been growing since the 1990s (Ellström, 2011; Kyndt et al., 2013; Poell & van Woerkom, 2011). This growing interest is centered in particular on the necessity of continuous learning (Billett, 2008; Gijbels et al., 2010; Molloy & Noe, 2010; Tynjälä, 2008) and subsequently also lifelong employability (Manuti et al., 2015). Prevailing theories of work-related learning emphasize the social dimension (Billett, 2002; Engeström, 2001; Eraut, 2000; Lave & Wenger, 1991).

Any kind of learning in the workplace implies an actual or hypothetical interaction with the work environment. Thus, learning does not occur isolated from others, but instead is a social process, which ─ in the sense intended by Vygotsky (1978) ─ is mediated by the environment. Workplace learning is inherently social (Billett, 2001a, 2004; Ellinger & Cseh, 2007; Eraut, 2000, 2004; Lave & Wenger, 1991; Marsick et al., 2017; Poell & van Woerkom, 2011; Rausch, 2013; Tynjälä, 2008) and one central aspect are social interactions (Collin & Valleala, 2005; Marsick et al., 2017; Rozkwitalska, 2019; Warhust & Black, 2015). By social interactions we refer to meaningful processes of verbal exchange between at least two people. Social interactions are particularly important for workplace learning within VET, in which social interactions with other people are core elements (Billett, 2010; Mikkonen et al., 2017). In addition, many social interactions of VET students are characterized by knowledge asymmetries, as they take place, for example, with colleagues or superiors. Moreover, in his typology of early career learning processes and activities, Eraut (2007) also emphasizes the importance of social interactions for workplace learning, especially for novices.

Although plenty of studies addressing workplace learning have focused on the social context of workplace learning, like for example communities of practice (Kirkman et al., 2013), learning networks (Melo & Beck, 2015), interpersonal relationships (Carmeli et al., 2009), leaderships styles (Froehlich et al., 2014), group learning and team learning (Gil & Mataveli, 2017; Raes et al., 2015; Watzek et al., 2019), help-seeking behavior after making an error (Grohnert et al., 2019) or social fun activities (Tews et al., 2017), to date, only a few studies have investigated the relationship between social interactions and informal learning in the workplace more deeply. In addition, most of the conducted studies were global qualitative studies or questionnaire studies with only a few items on social interactions, although micro-analyses of social interactions near the process are especially promising (Tschan et al., 2004). The limited number of studies that do exist identified, for example, interaction processes that supported workplace learning (Collin & Valleala, 2005) or learning experiences from mono- and intercultural interactions in the workplace (Rozkwitalska, 2019). In the VET context, social interactions were also addressed in broader studies that examined general facilitating factors for trainees’ workplace learning (Virtanen & Tynjälä, 2008; Virtanen et al., 2014). To our knowledge, there is neither a study that takes social interactions’ situational characteristics into account when investigating workplace learning nor a study that explicitly examines social interactions’ learning potential in the context of VET.

Furthermore, emotions (Benozzo & Colley, 2012; Hökkä et al., 2020) and personality traits (Cerasoli et al., 2018; Kyndt et al., 2013; Noe et al., 2014; Rintala et al., 2019) were identified as affecting learning in the workplace as well. Emotions are “an inevitable part of all workplace learning” (Beatty, 2011, p. 341) and “always colour learning” (Benozzo & Colley, 2012, p. 307). Personality traits are basic tendencies that impact a person’s thoughts, feelings and actions (McCrae & Costa, 1996). One of the most significant and widely used concepts in this field are the Big Five personality traits (Barrick & Mount, 1991; Li & Armstrong, 2015; Major et al., 2006), which include the five traits neuroticism, extraversion, openness to experience, agreeableness, and conscientiousness (McCrae & Costa, 1987, 1996, 1999). There is some empirical evidence that these traits can affect informal learning in the workplace (Li & Armstrong, 2015; Noe et al., 2013; Simmering et al., 2003; Takase et al., 2018). Although, as outlined, while there is some evidence on the influence of emotions as well as personality on workplace learning, only some of the studies were conducted in the context of social processes or contain social aspects. Moreover, the great majority of these studies was not conducted in the VET context.

Thus, based on data from a diary study, the present secondary analysis considers the hierarchical structure of the underlying data and addresses the influence of social interactions’ situational characteristics, emotions, and personality traits on learning from VET trainees' social interactions in the workplace. First, we will provide an overview of the theoretical constructs and related empirical work. The method section comprises details on the participants, the study design, the measures, and the analytical approaches. In the result section, we will present the test statistics. Finally, the results and applied methods are discussed, and we will offer an outlook for future research in this field as well as practical implications.

## Theoretical Framework and Related Research

### Workplace Learning and Social Interactions at Work

There are a lot of different definitions for workplace learning (Manuti et al., 2015; Tannenbaum et al., 2010). A commonly used definition describes workplace learning as processes that lead to the construction of new skills and competencies through work (Billett, 2001b; Eraut, 2000; Harteis et al., 2008). In general, workplace learning includes both formal and informal learning activities (Eraut, 2000, 2004; Janssens et al., 2017; Rintala et al., 2019; Schürmann & Beausaert, 2016). Eraut (2000) lists several central characteristics of a formal learning situation. These are a predefined learning framework, that is some kind of organized, predescribed learning goals, the presence of a trainer or teacher, and the receipt of a credit or qualification. In contrast, informal learning can be categorized by the absence of these features. Informal learning is unintended, unstructured and opportunistic, implicit and takes place in the absence of a designated teacher or trainer (Eraut, 2004). Huge parts of workplace learning take place informally (Eraut, 2010), as only a certain amount of occupational action knowledge and competence can be learned through formal learning activities (Dehnbostel, 2009).

Social interactions play a significant role in informal workplace learning. By social interactions we refer to meaningful processes of verbal exchange between at least two people. In general, social interaction of any kind may contribute to satisfying the need for relatedness as introduced within the Self- Determination Theory of motivation (Deci & Ryan, 1985). In this vein, Tschan et al. (2004) found that the frequency and perceived quality of interactions predicted affective commitment and job satisfaction in a new job. This may be all the more important for trainees since the other basic needs postulated within the Self-Determination Theory (Deci & Ryan, 1985), the need for competence and the need for autonomy, are largely unmet for newcomers in the workplace. Beyond relatedness, work-related interaction may also be conducive to the acquisition of competence and, thus, satisfy the need for competence in the long run. Following on from this, Bandura (1971) already situated learning in a social context within his Social Learning Theory and Situated Learning Theory emphasizes it as well (Lave & Wenger, 1991).

Although only a few studies were conducted in this context, their results indicate that social interactions can in fact have a positive impact on workplace learning. For instance, different clinical social spaces were found to be relevant for nurses’ workplace learning related to social interactions (Bono et al., 2007), and Rozkwitalska et al. (2017) and Rozkwitalska (2019) identified workplace learning as a frequent outcome of both, mono- and intercultural workplace interactions. Mulder (2013) revealed several significant correlations between feedback content characteristics and informal learning activities. Moreover, some significant relationships with informal learning activities were found for characteristics of the feedback delivery as well as for the perceived support for using the feedback. Daniels et al. (2009) identified as part of their experience sampling study that discussing problems with others to solve problems is a significant positive predictor of hourly learning assessed at the same time. Furthermore, it was shown that some of the most frequent informal workplace learning activities employees engage in are talking and collaborating with others as well as asking for and receiving feedback. In line with these findings, feedback, support and interacting with colleagues and supervisors were identified as important drivers for informal learning activities (Schürmann & Beausaert, 2016). In addition, Collin and Valleala (2005) revealed three main social situations at work that include interactions and foster learning in the workplace. These were 1) constant efforts to guarantee interaction and maintaining a sociable atmosphere and equality, 2) the production of categories, for example regarding customers, colleagues or work tasks resulting in categories knowledge, and 3) networked and situationally driven problem-solving.

Further studies identified feedback (Ellinger & Cseh, 2007; Janssens et al., 2017; Koopmans et al., 2006; Kyndt et al., 2009; Rausch, 2013; Takase et al., 2018), assistance from others (Ellinger & Cseh, 2007; Rausch, 2013), communication (Ellinger & Cseh, 2007; Janssens et al., 2017; Koopmans et al., 2006; Moon & Na, 2009; Warhust & Black, 2015), cooperation (Janssens et al., 2017), access to knowledge acquisition and information (e.g., participating in work groups or in conferences or workshops) (Janssens et al., 2017; Raes et al., 2015), (informal) coaching (Janssens et al., 2017; Kyndt et al., 2009; Warhust & Black, 2015), reflection (e.g., being asked for feedback by colleagues) (Janssens et al., 2017), informal networking with colleagues (Warhust & Black, 2015), asking questions (Ellinger & Cseh, 2007; Koopmans et al., 2006; Raes et al., 2015), constructive conflict (Raes et al., 2015), role playing (Ellinger & Cseh, 2007) as well as talking things through (Ellinger & Cseh, 2007) as being positively related to informal workplace learning. In the context of VET, discussions with employees (Virtanen & Tynjälä, 2008) as well as the availability of individual guidance and guidance concerning trainees’ development and assessment (Virtanen et al., 2014) were found to be related to trainees’ workplace learning.

Moreover, some studies have taken social interactions’ situational characteristics into account but only a few of them were conducted in the learning context. Previously addressed interaction characteristics were, for example, frequency and duration (Marlow et al., 2018; Matic et al., 2014; Noguchi-Watanabe et al., 2021; Tschan et al., 2004; Weijs-Perrée et al., 2020), regularity (Eddy et al., 2006), formality (Eddy et al., 2006; Matic et al., 2014), quality (Marlow et al., 2018), speech activity (Matic et al., 2014; Tschan et al., 2004), openness (Jeon & Kim, 2012), spatiality (Matic et al., 2014; Weijs-Perrée et al., 2020), initiation (Eddy et al., 2006; Kirmeyer, 1988; Tschan et al., 2004), participants (Eddy et al., 2006; Kirmeyer, 1988; Weijs-Perrée et al., 2020), aspects of the relationship between them (Eddy et al., 2006), face-to-face vs. at distance (Eddy et al., 2006) or interaction content and purpose (Eddy et al., 2006; Kirmeyer, 1988; Marlow et al., 2018; Tschan et al., 2004; Weijs-Perrée et al., 2020). Regarding the workplace learning context, it was shown, for example, that effective interactions were more likely mutually initiated and less likely initiated by a third party. In addition, the involvement of a direct supervisor and a more mandatory interaction more likely resulted in a lower effectiveness (Eddy et al., 2006). Furthermore, Jeon and Kim (2012) investigated open communication as a characteristic on the organizational and team level and found it to be significantly positively related to learning through interaction with peers.

### Personality, Emotions and Learning from Social Interactions

In addition to social interaction characteristics, personality traits and emotional experience can influence informal learning as well. Several personality traits like the Big Five personality traits, self-efficacy and goal orientation were found to influence informal workplace learning significantly positively (Cerasoli et al., 2018; Jeong et al., 2018; Kyndt et al., 2013; Noe et al., 2014; Rintala et al., 2019). For our study, we expect the Big Five personality traits (McCrae & Costa, 1987, 1996, 1999) to be particularly relevant, as they are related to interactions with others in the workplace (Mount et al., 1998).

The Big Five personality traits are an “empirical generalization about the covariation of personality traits” (McCrae & Costa, 1999, p. 139) and relatively time-stable during adult life (McCrae & Costa, 1996, 1999). A person’s specific trait profile influences his or her feelings, thoughts and actions (McCrae & Costa, 1996). The Big Five comprise the five traits of neuroticism, extraversion, openness to experience, agreeableness, and conscientiousness (McCrae & Costa, 1987, 1996, 1999). Persons high in neuroticism are characterized as being insecure, self-conscious, temperamental, and worrying. Furthermore, negative affect is central to neuroticism. This includes, for instance, feelings of depression, anger, anxiety and embarrassment (McCrae & Costa, 1987). Persons high in extraversion are described as being friendly, sociable, affectionate, and fun loving. The trait of openness to experience can be described with the adjectives imaginative, original, daring and broadly interested. It is further reflected in fantasy, feelings, ideas and aesthetics (McCrae & Costa, 1987). Agreeable people are sympathetic, helpful, cooperative and kind (McCrae & Costa, 1987; Saucier, 1994), while conscientious people are generally more scrupulous, dutiful, self-disciplined, ambitious and hardworking (McCrae & Costa, 1987). Tschan et al. (2004) found only very small effects of extraversion and social competences on the frequency and quality of interactions recorded in their diary study with 54 young professionals. Nevertheless, the expectation of an effect still appears plausible.

Concerning the Big Five personality traits and workplace learning, Noe et al. (2013) found significant positive correlations between all Big Five traits and informal learning, which included aspects of learning from oneself, learning from others and learning from non-interpersonal sources. However, when included in the regression analysis, they did not significantly predict informal learning. In their study on experiential learning styles according to Kolb (1984), Li and Armstrong (2015) identified extraversion as a significant positive predictor of learning from concrete experience (CE) and active experimentation (AE) and as a significant negative predictor of learning from reflective observation (RO) and abstract conceptualization (AC). Furthermore, agreeableness and conscientiousness predicted learning from CE significantly negatively. In another study, conscientiousness was also significantly positively related with postfeedback development at the ten percent significance level (Simmering et al., 2003). Moreover, Takase et al. (2018) found extraversion, conscientiousness and openness to experience to be significantly positively related to overall workplace learning, composed of learning from practice, learning from feedback, learning from training, learning from others and learning from reflection. Extraversion was also significantly positively related to all facets of workplace learning, while conscientiousness was significantly positively related to all facets but learning from others. Openness to experience showed significant positive relationships with learning from practice and learning from reflection. In addition, in a subsequent regression analysis, results yielded that extraversion and conscientiousness were both positive predictors of overall workplace learning as well.

Three out of four presented studies to some degree include informal learning related to social interactions. However, Noe et al. (2013) include it as one of three learning aspects summarized in a general variable addressing informal workplace learning. Regarding the study by Simmering et al. (2003), it is not entirely clear whether the participants got their feedback in solely written form or with additional feedback discussions, for example, and the development activities again include various aspects and sources of informal learning. Based on the presented empirical results and theoretical considerations, we expect some relations of the Big Five and informal learning from social interactions. However, due to the explorative nature of the research we do not formulate concrete hypotheses.

In accordance with the theoretical considerations of Noe et al. (2013), it may be likely that more agreeable people are more inclined to ask other people for help and generally engage in more frequent conversations as they are friendlier and more cooperative (McCrae & Costa, 1987; Saucier, 1994). In addition, we can imagine that because of this trait, these people also have quite good relationships with their colleagues and superiors, which makes them easier to approach and other people more willing to help. Beyond the empirical findings already presented on the influence of extraversion on workplace learning, in our opinion it may be possible that more extraverted people being sociable (McCrae & Costa, 1987) are also more likely to initiate and participate in conversations and that they are more likely to ask questions. Because extraverts like to socialize, they may tend to ask a person rather than use another source of help when they have a problem or question. All of these aspects may promote learning from social interactions (see also Noe et al., 2013).

Conscientiousness may be related to learning from social interactions as well, as people with this trait are generally more ambitious and scrupulous (McCrae & Costa, 1987) which may motivate them to seek help when faced with a problem or question (e.g., by asking other persons). Empirical evidence by Takase et al. (2018) may point to the expected relationship regarding extraversion and conscientiousness. Openness to experience is related to a broad interest in different things (McCrae & Costa, 1987). This may lead to people being very open-minded and interested in social interactions (McCrae & Costa, 1987), which could also have a positive effect on learning from them. People high in neuroticism are generally more insecure, affecting social interactions and subsequent learning outcomes as well (McCrae & Costa, 1987). For example, individuals with high neuroticism scores might be less confident to initiate and participate in social interactions. In addition, they might avoid asking questions and the associated acknowledgement of a lack of knowledge due to their uncertainty. As high neuroticism goes along with negative emotions (McCrae & Costa, 1987), these could also impact learning in different directions, something which will be discussed in more detail in the next section. Thus, we can imagine that all Big Five traits have an impact on learning from social interactions, which would at least be consistent with the correlational results of Noe et al. (2013).

Kleinginna and Kleinginna (1981) list 92 different definitions of emotions in the psychological literature and derive a comprehensive definition from them. According to them, emotion “is a complex set of interactions among subjective and objective factors, mediated by neural/hormonal systems, which can (a) give rise to affective experiences such as feelings of arousal, pleasure/displeasure; (b) generate cognitive processes such as emotionally relevant perceptual effects, appraisals, labeling processes; (c) activate widespread physiological adjustments to the arousing conditions; and (d) lead to behavior that is often, but not always, expressive, goal-directed, and adaptive” (Kleinginna & Kleinginna, 1981, p. 355). Research on emotions usually focuses on the subjective experience component. Furthermore, emotions are often considered to have a state and a trait component. While the trait component comprises stable individual differences in emotional experiences, the state component refers to transient episodes of emotional experiences or deviations in emotional responsiveness from the baseline (Nett et al., 2017; Watson & Clark, 1994).

Focusing on the subjective and the state component, Russell (1980) assumes in his circumplex model that emotions can be represented in one plane from a combination of the horizontal dimension *pleasure – displeasure* and the vertical dimension *arousal – sleep*. The first dimension is also referred to as valence, the latter as arousal (Feldman Barrett, 1998; Russell, 1980). A classification of emotions based on these two dimensions leads to a circular arrangement of them in one plane within the circumplex model (Russell, 1980). Within the scope of the Affective Events Theory (Weiss & Cropanzano, 1996) work related events are regarded in particular as the cause of emotional experiences in the workplace that again influence behavior and work attitudes. Furthermore, the Control-Value Theory (Pekrun, 2006) emphasizes the importance of achievement emotions on learning through motivational and cognitive mechanisms (Pekrun & Perry, 2014). Any emotion can be more or less useful for learning. Therefore, it would be inadequate to expect positive emotions to provoke learning and negative emotions to prevent learning (Pekrun & Perry, 2014). Although the Control-Value Theory refers primarily to the school context, its outlined general assumptions also hold for learning processes within the work context.

Several studies found emotions to influence informal workplace learning (Benozzo & Colley, 2012; Hökkä et al., 2020), but only some were conducted in the context of social processes. Daniels et al. (2009), for example, identified in their experience sampling study significant positive correlations of hourly learning with momentary activated pleasant affect and discussing problems to solve problems as well as momentary anxious affect with discussing problems to solve problems. Reio and Callahan (2004) found significant positive correlations of a modified version of the Workplace Adaptation Questionnaire, representing socialization-related learning in the workplace, with state anger, state curiosity and trait curiosity. In addition, the results of two subsequent path models yielded that state curiosity and trait curiosity affected socialization-related learning significantly positively. Moreover, Sebrant (2008) investigated nurses’ workplace learning in a qualitative study and the results showed that envy between two groups of nurses led to less cooperation and learning from each other.

Altogether, there is a deep theoretical foundation and rich empirical evidence for the significance of social interactions for workplace learning as well as the influence of personality traits and emotions. However, only a few studies address the influence of personality traits and emotions on workplace learning in a social context and none of them were conducted within VET. Based on the theoretical considerations and empirical evidence as well as the shortcomings of previous research, we address the following three research questions:

- RQ1: How do social interactions’ situational characteristics affect self-perceived learning from these interactions?
- RQ2: How do emotional experiences affect self-perceived learning from social interactions in the workplace?
- RQ3: How do personality traits affect self-perceived learning from social interactions in the workplace?

## Method

To investigate the above research questions, a diary study with a preceding questionnaire was conducted. Data was analyzed using multilevel analysis.

### Participants

To address the research questions, we conducted a diary study with 50 trainees within the German “Dual System” of vocational education and training (VET). The trainees were employed at a medium-sized utility company in Germany with 2,500 employees, amongst them 175 trainees, of which 50 participated in our study. They were at different stages of their apprenticeship programs and assigned in different departments, which is typical for dual apprenticeship. The mean age was 18.2 (SD = 1.04; min = 16; max = 21), 29 participants were female and 21 were male. A total of 22 of them were trainees in commercial trades (e.g., industrial management assistant; German: “Industriekaufmann/-frau”) and 28 were trainees in technical trades (e.g., industrial mechanic; German: “Industriemechaniker/in”).

### Procedure

A semi-standardized diary was applied to collect data in situ and to avoid the typical memory biases of retrospective measures such as questionnaires and interviews (Bolger et al., 2003; Ohly et al., 2010; Rausch, 2014). During the diary period, which comprised ten working days, the participants were instructed to record five typical social interactions at work every day. Considering school attendances (usually 1.5 days a week), holidays, illness or work-related hindrances, the diary period was set to four weeks. The participants were asked to record the interaction as soon as possible or only a few minutes after the interaction had occurred. In the context of this study, we were interested in interactions they had with trainers, supervisors, other trainees and so forth in their working day. Before the diary period, a workshop was conducted to clarify the term “social interaction” and to familiarize the participants with the diary. Before and after the diary period, the participants completed a self-report questionnaire, one of which included scales on the Big Five personality traits.

### Measures

#### Semi-standardized diary

Most of the items in the diary were standardized in that they offered a list of possible characteristics to choose from or a statement that had to be rated on a given scale. This was to ensure that an entry required only a minimum of effort. The diary was implemented as a paper-and-pencil version and was to be deposited in sealed boxes after the diary period.

To gain information on the interaction content, one item required the participants to choose from a list of possible contents for the social interaction (multiple selection): (a) an actual task demanded cooperation; (b) a concrete problem / an exception popped up, (c) instruction for new procedures that were unknown before; (d) planning / coordination upcoming workflows; (e) receiving feedback on past performance; (f) general issues concerning my apprenticeship program; (g) small talk / gossip; (h) other content. Thereafter, the participants gave a short complementary verbal description of the social interaction’s context. Moreover, six situational characteristics had to be rated: (i) speech proportion (1 = *I hardly said anything* to 6 = *I talked all the time*); (ii) questions asked (1 = *I asked no questions at all* to 6 = *I asked a great many questions*); (iii) atmosphere (1 = *very tense* to 6 = *very open*); (iv) time pressure (1 = *very high* to 6 = *no pressure at all*); (v) instrumentality (1 = *not helpful at all* to 6 = *very helpful*); (vi) self-perceived learning (1 = *learned nothing at all* to 6 = *learned a great deal*). These characteristics were derived from the presented theory and empirical studies as well as own considerations.

In addition, the participants indicated their emotional states throughout the social interaction. Eight emotional states were arranged according to common circumplex models of emotion with valence on the x-axis and arousal on the y-axis (Russell, 1980). The participants were asked to choose up to three out of eight emotional states they experienced during the social interaction and rate how strongly they experienced them (1 = *a little* to 3 = *very*). Each emotional state was described using three adjectives. These were (a) *motivated / delighted / curious*, (b) *confident / happy / glad*, (c) *contented / accepted / proud*, (d) *calm / even-tempered / daydreaming*, (e) *bored / dull / uninterested*, (f) *unhappy / gloomy / sad*, (g) *irritated / annoyed / angry* and (h) *nervous / worried / afraid*. Emotional states that were not chosen were coded with zero.

On average, the trainees kept the diary on 9.7 days and recorded 41.5 interactions each, resulting in n = 2,077 recorded interactions. Participants that recorded less than 20 interactions were excluded from the analyses. After that, 43 participants who recorded n = 1,989 interactions were left. Of the 1,989 social interactions, 452 interactions occurred as (a) the actual task demanded cooperation; 259 interactions occurred due to (b) a concrete problem / exception; 307 social interactions were (c) instructions; 423 social interactions referred to (d) the planning or coordination of upcoming workflows; in 108 interactions the trainees (e) received feedback; 198 interactions included (f) general issues concerning the apprenticeship program; 269 social interactions contained (g) small talk or gossip and 349 interactions were classified as (h) other content. Multiple assignments were possible. A total of 16.2 percent of social interactions were allocated to more than one content type. Table 1 provides some examples of the verbal description of social interactions out of the trainees’ diaries that were allocated to the different content types from (a) to (h).

**Table 1:** Examples of every content type for social interactions (multiple assignments were possible)

Content type	n	Examples from diary entries
(a) Actual task demanded cooperation	452	- The installation of a streetcar front windscreen - Calling a supplier, because questions arose regarding the invoice sent to us. According to our records, the invoice was already paid
(b) concrete problem / exception	259	- Changing the drill head - I didn’t know where to put the mail
(c) instruction	307	- Procedure for feeding electricity into the grid (photovoltaic systems) - Get circuit diagrams of the subway explained and asked appropriate questions about it
(d) planning / coordination upcoming workflows	423	- Planning of today’s tasks - Changes in my activities—new task assigned, which was more urgent in the moment
(e) receiving feedback	108	- Appraisal session with Mr. XY about past training phase - Praise for task well done
(f) general issues concerning apprenticeship program	198	- It was discussed when I want to take leave in the department - Intermediate examination discussed
(g) small talk / gossip	269	- Work colleague getting married received a gift from the department with congratulations - It was about a television program that ran the night before
(h) other content	349	- We studied for the presentation at school and thought about the schedule - Showing the intern the location

To investigate the research questions, only work-related interactions were included. These were the interaction categories (a) actual task demanded cooperation, (b) a concrete problem / an exception, (c) instruction, (d) planning / coordination upcoming workflows and (e) receiving feedback. 1,328 social interactions with these contents were reported by the participants. Little’s MCAR-Test indicated that the missing values were not missing completely at random (Chi-square = 703.2782, df = 270, p =  < 0.001). We assumed that the missing data mechanism is missing at random (MAR) (Newman, 2014) and imputed the missing data by using the R package *mice* (van Buuren & Groothuis-Oudshoorn, 2011). As recommended by Grund et al. (2018), we generated 20 imputations for the missing values.

#### Self-report questionnaire

To measure the Big Five personality traits, we administered the German version of Saucier’s (1994) “Big Five Mini-Markers” by Weller and Matiaske (2009). Sample adjectives for neuroticism are moody and jealous, for extraversion talkative and extraverted, for openness creative and intellectual, for agreeableness sympathetic and warm and for conscientiousness organized and practical. These mini markers consist of a list of 40 adjectives that are rated on a seven-point Likert-scale from 1 = *extremely inaccurate* to 7 = *extremely accurate*. The Cronbach’s α were calculated for Extraversion (α = 0.80), Neuroticism (α = 0.80), Conscientiousness (α = 0.80), Agreeableness (α = 0.69) and Openness (α = 0.50). The first three values are satisfactory (Streiner, 2003).

Table 2 shows means, standard deviations and correlations between the main study variables for the n = 43 participants and the n = 1,328 social interactions included in the regression analysis.

**Table 2:** Means, standard deviations and correlations between study variables

Variable	M	SD	M	SD	1	2	3	4	5	6	7	8	9	10	11	12	13	14	15	16	17	18	19	20
Big Five																								
1. Extraversion	4.92	0.88	-	-	-	–.03	.04	.27	.33*	–.19	.05	–.05	–.03	.35*	.19	.45**	.05	–.23	.01	.14	.30	–.10	–.08	.11
2. Neuroticism	2.74	0.89	-	-	-	-	–.41**	–.06	–.00	.23	.04	.14	.23	–.04	.01	–.20	.16	.29	.02	–.20	–.29	.10	–.23	.16
3. Conscientiousness	5.89	0.72	-	-	-	-	-	.20	.29	–.36*	–.28	–.14	–.26	.14	.04	.18	–.33*	–.17	–.26	.16	.21	–.10	.17	–.37*
4. Agreeableness	6.12	0.49	-	-	-	-	-	-	.16	–.02	–.02	.22	.32*	.16	–.03	.19	–.03	.22	–.08	.03	.04	–.40**	–.03	.06
5. Openess	5.01	0.56	-	-	-	-	-	-	-	–.34*	–.46**	–.39**	–.51***	.28	.01	.30*	–.33*	.09	.19	.20	.28	.04	.04	.12
Emotional experience																								
6.nervous / worried / afraid	0.15	0.23	0.13	0.46	–.11***	.12***	–.14***	–.00	–.12***	-	.74***	.68***	.59***	.10	.12	–.03	.41**	.23	.15	–.20	–.21	.45**	–.02	.40**
7. unhappy / gloomy / sad	0.06	0.16	0.04	0.26	.00	.03	.13***	–.02	–.19***	.29***	-	.80***	.69***	.14	.38*	.18	.62***	–.10	–.01	.02	.06	.22	–.00	.24
8. irritated / annoyed / angry	0.10	0.18	0.09	0.40	–.03	.09***	–.05	.07**	–.11***	.22***	.44***	-	.84***	.06	.25	.05	.40**	.14	–.19	–.01	–.11	.13	–.03	.16
9. bored / dull / uninterested	0.11	0.19	0.10	0.38	–.04	.12***	–.09**	.13***	–.19***	.08**	.18***	.40***	-	.05	.23	.04	.47**	.26	–.20	–.09	–.12	–.01	–.08	.20
10. motivated / delighted / curious	1.74	0.54	1.71	1.16	.17***	–.03	.11***	.06*	.19***	–.10***	–.12***	–.21***	–.21***	-	.47**	.61***	–.07	–.06	.15	.38*	.46**	.20	–.07	.25
11. contented / accepted / proud	0.64	0.56	0.60	1.01	.12***	.02	.05	.02	.05	–.08**	.01	–.04	–.05	.17***	-	.49***	.31*	–.00	–.08	.34*	.37*	.05	–.01	.20
12. confident / happy / glad	1.34	0.62	1.36	1.21	.25***	–.09**	.12***	.06*	.21***	–.17***	–.08**	–.16***	–.18***	.19***	.17***	-	.20	–.18	–.05	.43**	.70***	.07	.14	.10
13. calm / even-tempered / daydreaming	0.53	0.42	0.48	0.82	–.03	.07**	–.11***	–.04	–.16***	–.05	.03	–.02	.05	–.16***	–.09***	–.13***	-	.11	–.08	.01	.08	.05	.05	.26
Characteristics of social interactions																								
14. interruption	0.14	0.14	0.12	0.32	–.12***	.10***	–.03	.05	.01	.06*	–.03	–.01	.09***	.02	–.02	–.02	.05	-	–.05	–.10	–.37*	.07	.14	.23
15. instrumentality	3.53	0.76	3.55	1.58	–.02	–.01	–.12***	–.03	.07*	.05*	–.01	–.08**	–.12***	.19***	.01	.04	–.06*	.05	-	–.03	–.04	.32*	.07	.52***
16. time pressure	5.24	0.84	5.27	1.18	.10***	–.15***	.13***	.03	.19***	–.20***	–.05	–.10***	–.08**	.16***	.14***	.19***	.03	–.08**	.02	-	.63***	–.17	–.21	.13
17. atmosphere	5.18	0.61	5.21	0.99	.21***	–.16***	.13***	.02	.24***	–.21***	–.07**	–.22***	–.17***	.23***	.15***	.33***	.04	–.12***	–.01	.36***	-	–.12	–.03	–.03
18. questions asked	2.91	0.62	2.89	1.25	–.03	.06*	–.02	–.18***	.06*	.12***	.06*	.01	–.05	.18***	–.02	.04	–.06*	.07*	.42***	–.09**	–.04	-	.40**	.33*
19. speech proportion	3.04	0.54	3.05	1.02	–0.05	–.07*	.09**	–.04	.03	.05	.01	.03	–.03	–.02	.11***	.10***	–.08**	.00	.04	–.11***	.02	.28***	-	–.04
20. self-perceived learning	2.30	0.75	2.22	1.55	–.03	.08**	–.12***	.03	.09***	.05	.02	–.03	.01	.25***	.04	.07*	–.02	.13***	.54***	.06*	–.01	.38***	–.07*	-

Note. Means and standard deviations at the person level are displayed in column 1 and 2; means and standard deviations at the day level are displayed in columns 3 and 4; Correlations above the diagonal refer to person level data (level 2) (*N* = 43), with day level variables aggregated at the person level. Correlations below the diagonal refer to day level diary data (level 1) (n = 1,328)

*p < 0.05, ** *p* < 0.01, ***p < 0.001

### Multilevel Analysis

As the diary data is nested within persons, the data are analyzed by means of multilevel analysis (Hox et al., 2018; Snijders & Bosker, 2012). Multilevel analysis is a statistical approach for datasets with nested sources of variability (Snijders & Bosker, 2012). It aims at explaining variance sources at different levels of analysis (Hoffman & Rovine, 2007). As a rule of thumb, to conduct a multilevel analysis, at least 30 groups on the highest level should be available to reliably estimate the coefficients and standard deviations (Maas & Hox, 2005). This precondition is fulfilled by the present dataset. Although the data of the present analyses are multiple observations nested in persons, it is not necessary to analyze the data as longitudinal data because the intra-individual variation in social interactions over four weeks is not considered a function of time (Enders & Tofighi, 2007; Nezlek, 2001). Predictors on level 2 were centered at the grand mean, predictors on level 1 on the group mean (Enders & Tofighi, 2007). To control for the baseline level of emotional experience and the baseline level of interaction situational characteristics for every trainee, the group mean, and therefore the mean for each trainee is used as a supplementary control variable. These refer to the trait component of emotional experiences and interaction characteristics.

The presented research questions are tested in a series of multilevel models using the free software R. First the control variables are included (Model 1), then the characteristics of the social interaction (Model 2), after that the emotional experience (Model 3) and in Model 4 we included the Big Five personality traits. All models were calculated as means-as-outcomes models. To check for the improvement of model fit, the ∆-2*log statistics are calculated. The number of *df*s resulted from the number of new predictors added. The Pseudo-R^2^ value is calculated according to the formula proposed by Snijders and Bosker (2012).

## Results

Before the investigation of the research questions, we calculated the intraclass correlation coefficient (ICC), using the intercept-only model. The ICC for self-perceived learning from social interactions is 0.186, indicating that 18.6 percent of the variance can be explained by differences in Level 2 and therefore by differences between the persons. Although the use of multilevel models is generally recommended for nested data, this ICC value clearly advocates multilevel modeling (Musca et al., 2011; Nezlek, 2008).

The analysis was started by computing the intercept-only model. In model 1, we included the control variables to control for the baseline level of emotional experiences and the general level of the social interactions’ characteristics for the single participants. Table 3 shows the results of all models. Model 1 fits the data better than the intercept-only model. The *baseline level of instrumentality* of social interactions (= Ø instrumentality) was a significant predictor for the self-perceived learning from social interactions (*B* = 0.473, *SEB* = 0.112, *p* < 0.001).

**Table 3:** Multilevel estimates for models predicting self-perceived learning from social interaction

	Null model			Model 1				Model 2				Model 3				Model 4			
	Estimate	SE	t	Estimate	SE	Beta	t	Estimate	SE	Beta	t	Estimate	SE	Beta	t	Estimate	SE	Beta	t
Intercept	2.2832	0.111	20.62***	-0.192	1.136		-0.169	-0.288	1.153		-0.250	-0.291	1.154		-0.252	-0.153	1.131		-0.135
Control variables																			
Ø nervous / worried / afraid				1.260	0.638	.427	1.976	1.065	0.645	.361	1.652	1.076	0.645	.365	1.677	1.168	0.656	.396	1.780
Ø unhappy / gloomy / sad				-2.089	1.129	-.490	-1.850	-1.709	1.142	-.401	-1.496	-1.739	1.143	-.408	-1.522	-1.569	1.271	-.368	-1.235
Ø irritated / annoyed / angry				-0.056	1.092	-.014	-0.051	-0.112	1.108	-.029	-0.101	-0.115	1.109	-.030	-0.103	-0.442	1.117	-.115	-0.395
Ø bored / dull / uninterested				0.846	0.831	.234	1.017	0.808	0.837	.224	0.965	0.823	0.838	.228	0.982	1.106	0.930	.307	1.189
Ø motivated / delighted / curious				0.062	0.214	.049	0.289	0.061	0.217	.049	0.283	0.062	0.217	.050	0.287	0.011	0.207	.009	0.052
Ø contented / accepted / proud				0.179	0.178	.146	1.009	0.163	0.180	.132	0.905	0.164	0.180	.134	0.913	0.216	0.170	.175	1.269
Ø confident / happy / glad				0.137	0.211	.123	0.650	0.127	0.214	.114	0.591	0.125	0.214	.112	0.584	-0.053	0.213	-.048	-0.248
Ø calm / even-tempered / daydreaming				0.468	0.270	.286	1.732	0.445	0.273	.272	1.632	0.449	0.273	.274	1.645	0.432	0.258	.264	1.671
Ø instrumentality				0.473	0.112	.527	4.216***	0.481	0.114	.536	4.204***	0.481	0.115	.536	4.199***	0.379	0.114	.422	3.322**
Ø time pressure				0.223	0.125	.273	1.781	0.212	0.128	.259	1.658	0.214	0.128	.262	1.673	0.240	0.120	.294	1.998
Ø atmosphere				-0.213	0.209	-.190	-1.019	-0.190	0.213	-.170	-0.889	-0.193	0.214	-.172	-0.904	-0.186	0.202	-.166	-0.922
Ø questions asked				0.151	0.178	.137	0.850	0.165	0.179	.149	0.922	0.164	0.179	.149	0.920	0.246	0.198	.222	1.241
Ø speech proportion				-0.170	0.171	-.134	-0.992	-0.174	0.173	-.137	-1.006	-0.170	0.173	-.135	-0.984	-0.141	0.173	-.112	-0.816
Characteristics of the social interaction																			
interruption								0.311	0.104	.220	2.982**	0.277	0.104	.196	2.667**	0.266	0.104	.188	2.562*
instrumentality								0.464	0.025	.451	18.248***	0.447	0.026	.435	17.512***	0.447	0.026	.435	17.516***
time pressure								0.040	0.038	.024	1.069	0.031	0.038	.018	0.812	0.030	0.038	.018	0.805
atmosphere								0.003	0.041	.002	0.084	-0.024	0.042	-.013	-0.561	-0.024	0.042	-.013	-0.563
questions asked								0.274	0.032	.215	8.516***	0.259	0.032	.204	8.078***	0.259	0.032	.204	8.079***
speech proportion								-0.175	0.036	-.109	-4.792***	-0.169	0.038	-.106	-4.605***	-0.170	0.037	-.106	-4.608***
Emotional experiences																			
nervous / worried / afraid												-0.109	0.081	-.032	-1.336	-0.108	0.081	-.032	-1.332
unhappy / gloomy / sad												0.008	0.149	.001	0.055	0.008	0.149	.001	0.056
irritated / annoyed / angry												-0.029	0.098	-.008	-0.296	-0.030	0.098	-.008	-0.301
bored / dull / uninterested												0.267	0.099	.066	2.708**	0.268	0.099	.066	2.717**
motivated / delighted / curious												0.167	0.034	.120	4.938***	0.167	0.034	.120	4.943***
contented / accepted / proud												-0.004	0.037	-.002	-0.095	-0.004	0.037	-.002	-0.096
onfident / happy / glad												0.042	0.032	.031	1.321	0.042	0.032	.031	1.328
calm / even-tempered / daydreaming												-0.047	0.046	-.024	-1.021	-0.046	0.046	-.024	-1.014
Big Five																			
Extraversion																0.100	0.106	.128	0.943
Neuroticism																-0.090	0.101	-.118	-0.901
Conscientiousness																-0.205	0.131	-.216	-1.565
Agreeableness																0.134	0.229	.069	0.584
Openness																0.284	0.201	.234	1.409
-2 * log	4771.665			4736.931				4171.490				4132.701				4125.829			
Diff -2*log				34.724***				565.440***				38.789***				6.872			
Δ df				13				6				8				5			
R^2^ Level 1				0.12				0.40				0.41				0.43			

^*^ p < 0.05 ** p < 0.01 ***p < 0.001

In model 2, we added the situational characteristics of the social interactions as predictors. The model fit further increased. The characteristics *interruption* (*B* = 0.311, *SEB* = 0.104, *p* < 0.01), *instrumentality* (*B* = 0.464, *SEB* = 0.025, *p* < 0.001) and *questions asked* by the trainee (*B* = 0.274, *SEB* = 0.032, *p* < 0.001) were identified to be positive significant predictors of self-perceived learning from social interactions. The characteristic *speech proportion*, however, was a significant negative predictor (*B* = -0.175, *SEB* = 0.036, *p* < 0.001).

In model 3, the emotional experiences during the social interactions were included as predictors. Again, the model fit improved and the emotional experiences *bored / dull / uninterested* (*B* = 0.267, *SEB* = 0.099, *p* < 0.01) and *motivated / delighted / curious* (*B* = 0.167, *SEB* = 0.034, *p* < 0.001) were positive significant predictors of the self-perceived learning from social interactions. Emotions’ baseline level did not have a significant influence. In model 4, the Big Five personality traits were included as predictors. However, the model fit did not improve significantly.

Regarding the standardized coefficients in model 3, the *baseline level of the instrumentality* of social interactions (*β* = 0.536) and the *instrumentality* of the current interaction (*β* = 0.435) were the strongest predictors of self-perceived learning from social interactions. Furthermore, there were moderate effects of *questions asked* (*β* = 0.204), the occurrence of an *interruption* (*β* = 0.196) and a low *speech proportion* (*β* = -0.106). Regarding emotional experiences, feeling *motivated / delighted / curious* was the strongest predictor (*β* = 0.120).

## Discussion

In this study, we investigated the effects of situational characteristics of social interactions, emotional experiences and personality on learning from these social interactions at work. Data from 43 trainees within the German dual system of vocational education and training (VET) were analyzed. These trainees recorded 1,328 work-related social interactions. A multilevel analysis was conducted to address three research questions on the influences of the characteristics of social interactions (RQ1), of emotional experiences (RQ2), and of personality traits (RQ3) on self-perceived learning from social interactions. RQ1 and RQ2 are addressed based on model 3 of the multilevel analysis because the inclusion of the Big Five personality traits (RQ3) in model 4 did not further improve the model fit.

RQ1 addressed the influence of situational characteristics of social interactions on self-perceived learning from these interactions. The results reveal that the *baseline level of instrumentality* (= Ø instrumentality in Table 3) of the social interactions, an *interruption* of the social interaction, the *instrumentality* of social interactions and *questions asked* are significant positive predictors of self-perceived learning from social interactions. The trainees’ *speech proportion*, however, is a significant negative predictor of learning. It seems plausible for novices that asking questions and listening to answers by more experienced co-workers is conducive to learning. In addition, this is in line with the findings of other studies that also identified asking questions as a behavior supporting learning (Koopmans et al., 2006; Raes et al., 2015).

Furthermore, not only does the perceived instrumentality of an interaction (i.e. the perception of how helpful the current interaction is) foster learning but also the baseline level of instrumentality (i.e. an individual’s general tendency to perceive interactions as instrumental for their work activities) fosters learning. This general tendency can be due to individual dispositions such as interest or a general openness, but it can also be caused by contextual factors such as particularly supportive colleagues. The positive influence of an interruption of the social interactions on self-perceived learning is surprising. Unfortunately, we do not have information on the type of interruption. It might be that a more experienced colleague explained a current work task when the interruption occurred. During this pause the trainee might have reflected on his or her understanding and might have thought about clarifying questions. However, it could also be that the explaining person was forced to continue with a current work task and the trainee learned from observing. Finally, the longer an interaction takes, the higher the probability that an interruption occurs, while the probability that there are opportunities to learn is also higher. In line with this, the duration of a social interaction and an interruption of the interaction are significantly positively related in our data (*r* = 0.26, *p* < 0.001).

RQ2 addressed the influence of emotional experience during social interactions on self-perceived learning from social interactions. Results reveal that feeling *motivated / delighted / curious*, that is states of high arousal and medium pleasure, as well as feeling *bored / dull / uninterested*, that is states of moderate displeasure and high sleepiness, have a significant positive influence on self-perceived learning from social interactions. Thus, according to our results, high levels at both ends of the continuum *arousal – sleep* seem to promote learning. Regarding emotions with high arousal, a lot of other studies found a positive influence of motivation on workplace learning as well (Cerasoli et al., 2018; Rintala et al., 2019; Tynjälä, 2013). In addition, Reio and Callahan’s (2004) results yielded a significant positive effect of state curiosity on socialization-related learning, which is in line with our results. The positive influence of states of moderate displeasure and high sleepiness is surprising. As such a state boredom usually has a negative impact on learning (Goetz & Hall, 2014). However, Nett et al. (2011) found that the “behavioral-approach” towards coping with boredom includes behaviors to change the situation, for instance, by asking for other tasks that are more interesting and challenging. That in turn could encourage learning. Another possible explanation is the assumption that boredom arises from being underchallenged (Csikszentmihalyi, 1988), which was found in first-year VET trainees by Nickolaus et al. (2009). Therefore, in our study, it could be that high-ability trainees who quickly understand what is discussed experience states of low arousal and lower pleasure rather than being challenged by possible further explanations and examples. Hence, these emotional states during the interactions would point to trainees’ already high competencies. We could not find a significant influence of emotions’ baseline level. Thus, in contradiction to, for example, Reio and Callahan (2004), we did not find a significant impact of emotional experiences’ trait component on learning.

RQ3 addressed the influence of the Big Five personality traits on self-perceived learning from social interactions. The inclusion of the Big Five in Model 4 did not improve the model fit. Therefore, it can be concluded that the Big Five have no significant influence on self-perceived learning from social interaction. However, they show several significant correlations with emotional experiences. It is possible that other personality traits that we did not include in our analysis are more important in this context. This could include, for example, zest (Noe et al., 2013). Looking at the standardized regression coefficients, results show that the strongest predictor is found in the situational characteristics of the interactions, that is the baseline level of instrumentality. Social interactions characteristics’ inclusion in the regression analysis also led to the largest increase in explained variance in self-perceived learning from social interactions. Including emotional experiences hardly increased the explained variance.

In summary, our results confirm social interactions’ potential to foster informal workplace learning as also found in a some prior studies (Bono et al., 2007; Daniels et al., 2009; Mulder, 2013; Rozkwitalska, 2019; Rozkwitalska et al., 2017; Schürmann & Beausaert, 2016). According to our results social interactions with a low speech proportion of trainees but in which they have the opportunity to ask questions, that include interruptions and that provoke emotional experiences of moderate displeasure and high sleepiness as well as states of high arousal and medium pleasure are conducive for self-perceived learning from social interactions. Furthermore, our research opens the avenue to explicitly include situational characteristics of social interactions into research. Regarding emotional experiences, there are very few studies on the effect of positive emotions on workplace learning (Hökkä et al., 2020). Together with these existing studies (Daniels et al., 2009; Owen, 2016; Rausch et al., 2015, 2017; Watzek et al., 2019) our research helps address this gap. Moreover, applying the diary method provided valuable insights into the situational characteristics of trainees’ everyday social interactions in the workplace. In addition, it meets Tschan et al.’s (2004) calls to study social interactions by means of micro-analyses and in natural settings.

## Limitations

Our study has several limitations. Firstly, reporting learning requires being aware of it. Implicit learning, however, often happens without one being aware of it (Eraut, 2000, 2004). Furthermore, learning was addressed with only one item in the diary. Hence, some aspects of informal learning may therefore not be evident in the diaries. In addition, keeping the diary might have fostered learning because completing the diary form also triggers reflection, so some aspects of learning could also be overreported. Moreover, the causality on interaction-level can be questioned. For instance, having learned something could affect the perceived instrumentality of an interaction or could lead to feeling motivated. A further limitation is the fact that for reasons of completeness we included the Big Five personality trait of openness into the analysis despite it having a very poor Cronbach’s alpha. However, it had no significant impact on learning from social interactions. Furthermore, we cannot be sure whether we included all relevant situational characteristics of social interactions in our research and we have not controlled for the personal relationship between the interaction partners, but this could certainly play a role in the perception of the interaction. Finally, the generalizability of the findings is limited as the sample is a non-probability convenience sample and thus generally not representative.

## Practical Implications and Future Research

Learning from social interactions in the workplace is considered a major source of informal workplace learning. Trainees’ learning from social interaction increases if the interaction is perceived as instrumental for future work activities. Furthermore, self-perceived learning increases with the amount of questions asked and with a smaller share of their own speech. In a nutshell, skilled workers should focus on relevant content to foster trainees’ learning, and trainees should ask questions and listen to their more experienced colleagues. Training companies should foster these kinds of interactions by acknowledging skilled workers’ engagement in instructing and guiding trainees and by granting them extra time to do so. Trainees should be encouraged to ask questions whenever something is unclear to them.

As the data were collected in this study in only one company and only with trainees, future research should be conducted in other companies in different contexts and industries and also with more experienced employees. By doing so, it would then be possible to compare the findings. This would be interesting, especially because of the rather surprising positive influence of emotional states of low arousal and lower pleasure and of an interruption in social interactions. Further research should also focus on social interactions’ situational characteristics to continue micro-analyses. In future investigations, data on the interaction content and the grade of trainees’ school learning certificate could be collected as we expect them to be illuminating. In addition, the relation between learning from social interaction and other sources of learning could be a very informative focus of subsequent research. Finally, against the background of COVID-19 and the increase in the amount of home office work, which will presumably remain in the future in at least a weakened form, it would be interesting to examine the influence of face-to-face social interactions versus digital interaction. Here, a focus could also be on whether the delivery mode serves as a moderator between the various potential influencing factors (e.g., characteristics of the social interaction, personality) and learning from social interactions.

## Data Availability

The dataset analyzed during the current study is available from the corresponding author on reasonable request.
